# How cigarette smoking may increase the risk of anxiety symptoms and anxiety disorders: a critical review of biological pathways

**DOI:** 10.1002/brb3.137

**Published:** 2013-03-26

**Authors:** Steven Moylan, Felice N Jacka, Julie A Pasco, Michael Berk

**Affiliations:** 1 Deakin University School of MedicineBarwon Health, Geelong, Victoria, Australia; 2 Department of Psychiatry, Melbourne UniversityParkville, Victoria, Australia; 3 NorthWest Academic Centre, Department of Medicine, The University of MelbourneSt Albans, Victoria, Australia; 4 Orygen Youth Health Research Centre, Centre for Youth Mental HealthParkville, Victoria, Australia; 5 The Mental Health Research Institute of VictoriaParkville, Victoria, Australia

**Keywords:** Anxiety, anxiety disorder, cigarette, epigenetic, inflammation, mitochondria, neurodevelopment, neurotransmitters, neurotrophins, nicotine, nitrosative stress, oxidative stress

## Abstract

Multiple studies have demonstrated an association between cigarette smoking and increased anxiety symptoms or disorders, with early life exposures potentially predisposing to enhanced anxiety responses in later life. Explanatory models support a potential role for neurotransmitter systems, inflammation, oxidative and nitrosative stress, mitochondrial dysfunction, neurotrophins and neurogenesis, and epigenetic effects, in anxiety pathogenesis. All of these pathways are affected by exposure to cigarette smoke components, including nicotine and free radicals. This review critically examines and summarizes the literature exploring the role of these systems in increased anxiety and how exposure to cigarette smoke may contribute to this pathology at a biological level. Further, this review explores the effects of cigarette smoke on normal neurodevelopment and anxiety control, suggesting how exposure in early life (prenatal, infancy, and adolescence) may predispose to higher anxiety in later life. A large heterogenous literature was reviewed that detailed the association between cigarette smoking and anxiety symptoms and disorders with structural brain changes, inflammation, and cell-mediated immune markers, markers of oxidative and nitrosative stress, mitochondrial function, neurotransmitter systems, neurotrophins and neurogenesis. Some preliminary data were found for potential epigenetic effects. The literature provides some support for a potential interaction between cigarette smoking, anxiety symptoms and disorders, and the above pathways; however, limitations exist particularly in delineating causative effects. The literature also provides insight into potential effects of cigarette smoke, in particular nicotine, on neurodevelopment. The potential treatment implications of these findings are discussed in regards to future therapeutic targets for anxiety. The aforementioned pathways may help mediate increased anxiety seen in people who smoke. Further research into the specific actions of nicotine and other cigarette components on these pathways, and how these pathways interact, may provide insights that lead to new treatment for anxiety and a greater understanding of anxiety pathogenesis.

## Introduction

Cigarette smoking is the single biggest contributor to death and morbidity worldwide (Gellert et al. 2012). Smoking rates are significantly higher in anxiety-disordered populations (Lasser et al. 2000; Tobias et al. 2008; Lawrence et al. 2010), and numerous studies support a relationship between cigarette smoking and psychiatric disorders (see review Dome et al. 2010). Three nonmutually exclusive models may explain the smoking–anxiety association (Moylan et al. 2012a). First, smoking may lead to increased anxiety; second, anxiety may increase smoking rates; or third, smoking and anxiety rates may both be influenced by shared vulnerability factor(s).

Evidence suggests that individuals with increased anxiety are more likely to smoke (Brown et al. 1996; Patton et al. 1998; Sonntag et al. 2000; Goodwin et al. 2005; Cuijpers et al. 2007; Swendsen et al. 2010). Multiple factors have been proposed to explain this, including use of cigarettes to reduce anxiety (i.e., self-treatment) and an increased susceptibility of those with anxiety in initiating smoking in response to peer pressure (Patton et al. 1998; Tjora et al. 2011). In addition, smoking appears to increase the risk of developing increased anxiety (Breslau and Klein 1999; Johnson et al. 2000; Isensee et al. 2003; Goodwin et al. 2005; Cuijpers et al. 2007; Pedersen and von Soest 2009). Potential explanatory models for this include the effects of smoking on neurotransmitters, neurobiology, respiratory health and autonomic control (Klein 1993; Niedermaier et al. 1993; Zvolensky et al. 2003; Zvolensky and Bernstein 2005; Preter and Klein 2008), in addition to effects on normal neurodevelopmental (Dwyer et al. 2008; Iniguez et al. 2009). Finally, numerous shared vulnerability factors have been identified that may increase the likelihood of both smoking and increased anxiety (Reichborn-Kjennerud et al. 2004; Hettema et al. 2005). For example, lower socioeconomic status is associated with both increased smoking behaviors (Schaap and Kunst 2009; Tjora et al. 2011) and anxiety (Kessler et al. 2005).

Despite the significant health impacts arising from the comorbidity between smoking and anxiety, the biological mechanisms underpinning this association have received less investigation than for other psychiatric disorders. The relationship between smoking and anxiety is complex as evidence supports that cigarette smoke can reduce anxiety in some smokers (see review Morissette et al. 2007). In addition, smokers often report increased anxiety post smoking cessation, although recent data conflict with this finding (Bolam et al. 2011; McDermott et al. 2013). There is also significant heritability in both anxiety expression and smoking behaviors.

Recent advances in understanding the etiology of mood and anxiety disorders support a key role for neurotransmitter systems, the immune system, oxidative and nitrogen stress (O&NS), mitochondrial dysfunction, neurotrophins (NTs) and neurogenesis, and epigenetic effects in pathogenesis (Berk et al. 2011; Moylan et al. 2012b). All of these systems are affected by exposure to cigarette smoke.

This review critically examines and summarizes the literature that has explored how cigarette smoking may increase the likelihood of developing increased anxiety and anxiety disorders. In this review, we focus on relevant biological mechanisms (e.g., neurotransmitter systems, inflammation, oxidative and nitrosative stress, mitochondrial dysfunction, dysregulation of NTs and neurogenesis, and epigenetic effects) that potentially mediate how smoking may influence anxiety symptoms. Extensive literature has explored numerous psychological and social contributors to a relationship between anxiety and cigarette smoking. Readers interested in these pathways should consult the numerous excellent reviews available (Zvolensky et al. 2005; Morissette et al. 2007; Ameringer and Leventhal 2010). These reviews also contain discussions regarding anxiety definition and symptom structures of different anxiety disorders. Our aim with the discussion of these specific pathways was to provide a framework for future research that can be integrated with other theories of anxiety development, to hopefully lead to a holistic understanding of anxiety pathogenesis.

## Methods

For this narrative review, we searched biomedical databases PubMed, Embase, and PsycInfo in mid-2012 using terms “anxiety”, “anxiety disorder”, “panic disorder”, “post traumatic stress disorder”, “obsessive compulsive disorder”, “generalized anxiety disorder”, “social phobia”, “inflammation”, “immune”, “tobacco”, “cigarette”, “smoke”, “nicotine”, “oxidative stress”, “nitrogen stress”, “mitochondria”, “neurotrophin”, “neurogenesis”, and “psychiatry” in various combinations to identify relevant papers for the outlined sections. We did not limit the search by year of publication, but did limit to English language publications. We were deliberately inclusive in identifying relevant papers due to the scant availability of research in some areas. All bibliographies of identified papers were searched for further relevant information. Once acquired, studies were grouped into the following headings: clinical studies, structural brain changes and clinical correlates, neurotransmitter systems, inflammation and cell-mediated immune activation, oxidative and nitrosative stress, mitochondrial function, NTs and neurogenesis, epigenetics and neurodevelopmental effects.

Where possible, we presented literature pertaining to individual anxiety disorder states (e.g., panic disorder [PD], post traumatic stress disorder [PTSD], generalized anxiety disorder [GAD] etc.). Where this was not possible (e.g., in discussion of studies pertaining to psychological stress or anxiety symptom scores, or in animal studies), we have presented results relating to “anxiety” in general.

## Clinical Studies

### Cigarette smoking as risk factor for anxiety disorders – epidemiological studies

In addition to many studies demonstrating a cross-sectional relationship between cigarette smoking and anxiety disorders, numerous population-based studies (Breslau and Klein 1999; Johnson et al. 2000; Isensee et al. 2003; Goodwin et al. 2005; Cuijpers et al. 2007; Chou et al. 2011) have demonstrated smoking as being prospectively associated with increased rates of anxiety disorders (see review Moylan et al. 2012a). Although most have utilized adult populations, some studies have demonstrated adolescent smoking as being associated with increased rates of some anxiety disorders (Johnson et al. 2000; Goodwin et al. 2005). For example, in a study utilizing data from the Oregon Adolescent Depression Project, the odds ratio of expressing PD at age 24 when comparing baseline (age 14–18) daily smoking to nondaily smoking was 5.1 (95% CI 2.4–10.5), which remained significant after controlling for other anxiety disorders and parental risk factors. Similarly, increased odds of numerous adult anxiety disorders (GAD: OR 5.53 [1.84–16.66] and PD: OR 15.58 [2.31–105.14]) and all anxiety disorders when grouped (OR 10.78 [1.48–78.55]) was discovered for baseline (age 14) heavy smokers (20+ cigarettes day) versus nonheavy smokers (1–19 cigarettes daily) in adolescence (Johnson et al. 2000). Breslau and Klein (1999) also demonstrated smoking as increasing the risk of first onset of both PD and panic attacks. In addition, nicotine dependence has been associated with increased odds of anxiety disorders (OR: 2.2 for men, 2.6 for women) (Breslau et al. 1991).

## Laboratory/Biological Studies

### Structural brain changes and clinical correlates

Cigarette smoking and anxiety disorders are both associated with structural brain changes. Cigarette smoking has been associated with diverse changes, including reduction in integrity of cerebral white matter microstructure (Gons et al. 2011), reduced prefrontal cortices (PFC) gray matter volumes (Brody et al. 2004; Zhang et al. 2011a), reduced gray matter volume or density in the anterior and posterior cingulate gyri (Brody et al. 2004; Gallinat et al. 2006), and reduced volume of frontal and temporal cortices and cerebellum (Gallinat et al. 2006) which may be consequence of direct toxic or adaptive effects. Importantly, these changes appear correlated with magnitude of cigarette exposure. For example, the measured volumes of frontal lobes, temporal lobes, and the cerebellum of smokers are inversely correlated with magnitude of life exposure to tobacco smoke, measured in pack years (*P* = 0.001) (Gallinat et al. 2006). In addition, pack years of smoking is inversely correlated with density of PFC gray matter (Brody et al. 2004). These changes overlap to some degree with neuroimaging changes observed in mood and anxiety disorders (Moylan et al. 2012b). Correlates of these structural changes may be associated with cognitive deficits as consequence of cigarette smoking (Durazzo et al. 2010), which have been repeatedly demonstrated in smoking populations (Richards et al. 2003; Nooyens et al. 2008; Peters et al. 2008; Sabia et al. 2012). For example, smoking is associated with reduced cognitive performance, and cognitive performance improves with increasing time since smoking cessation (Gons et al. 2011).

Individuals with anxiety disorders exhibit structural brain changes potentially resulting from illness related or secondary effects, although investigation in this area is still evolving (Damsa et al. 2009). Many studies have demonstrated the essential role of the amygdala, anterior insula, and anterior cingulate cortex in the key processing of fear conditioning and extinction, and potential role of the PFC structures as possible moderators of amygdala fear responses during extinction (Holzschneider and Mulert 2011). Studies have identified volumetric brain changes in patients with PD (Asami et al. 2008, 2009; Hayano et al. 2009), including reduction in the anterior cingulate cortex (Johnson et al. 2000; Asami et al. 2008), right ventromedial cortex and amygdala, bilateral insular cortex, occipitotemporal gyrus (Pedersen and von Soest 2009) and lateral temporal cortex (van Tol et al. 2010). In patients with GAD, volumetric assessment has produced inconsistent results including increased (De Bellis et al. 2000; Schienle et al. 2011) and decreased amygdala (Milham et al. 2005) volumes and alterations to the PFC (Schienle et al. 2011), which possibly relates to heterogeneity of samples used. Lifetime GAD has also been associated with reduced hippocampal volumes, an effect independent of major depressive disorder (Hettema et al. 2012). Functional studies have utilized various symptom provocation models for specific anxiety symptoms dependent upon the disorder being studied. Besides results in obsessive–compulsive disorder (OCD), where the predominant response is hyperactivity of the anterior cingulate cortex (Deckersbach et al. 2006), the majority of studies demonstrate hyperactivity of brain regions associated with the fear response (amygdala), and hypoactivity in areas thought to regulate the fear responses (e.g., anterior cingulate cortex, PFC) (Holzschneider and Mulert 2011).

Changes to white matter microstructure are present in both smokers and individuals with anxiety disorders. Cigarette smoking appears to influence the integrity of white matter (measured by change in fractional anisotropy [FA]); however, variables such as age and nicotine dependence appear to moderate this effect (Paul et al. 2008; Gons et al. 2011). In available studies, cigarette smoking is associated with increased measures of FA, although levels of FA are negatively correlated with cigarette exposure and nicotine dependence. For example, a study of adults (33.7 ± 7.9 years) by Hudkins et al. (2012) investigating white matter microstructure demonstrated that smokers exhibited higher FA in multiple white matter regions than age-matched controls, but that the magnitude of cigarette consumption and nicotine dependence was negatively correlated with FA. Higher FA in smokers was also shown in other studies (Jacobsen et al. 2007; Paul et al. 2008), although FA increased with lower levels of cigarette exposure (Paul et al. 2008). In a further study, levels of FA were lower in smokers than nonsmokers (Berk et al. 2011). Attempting to resolve these conflicting results, Hudkins et al. (2012) hypothesized that FA could be increased in smokers, particularly in adolescent smokers, due to the direct effects of nicotine stimulating glial proliferation and activity (Paul et al. 2008; Hudkins et al. 2012). This effect would be more pronounced in adolescence, as white matter proliferation is faster in adolescence than adulthood. As exposure to cigarette smoking continues through adult life, FA would decrease faster in smokers than nonsmokers, secondary to potential toxic effects of cigarette smoke, leading to lower FA overtime (Hudkins et al. 2012). Similar to findings in smoking, a number of studies have demonstrated altered white matter structural integrity across various anxiety disorders (for review see Ayling et al. 2012). For example, patients with PD demonstrated higher FA values in the left anterior and right posterior cingulate correlated with symptom severity (Han et al. 2008). Further, studies in patients with GAD demonstrated reduced FA in the uncinate fasciculus (Hettema et al. 2012) (connecting the amygdala and orbitofrontal cortex), a result also demonstrated in social phobia (SP) (Phan et al. 2009; Baur et al. 2011), and increased FA in the right postcentral gyrus (Zhang et al. 2011b). In PTSD, lowered FA has been found in areas including the left frontal gyrus, internal capsule, and midbrain (Kim et al. 2005; Schuff et al. 2011). Changes in integrity of white matter pathways connecting fear areas, including the uncinate fasciculus and corpus callosum, have been associated with trait anxiety states (Kim and Whalen 2009; Baur et al. 2011; Westlye et al. 2011).

We are aware of only one study assessing the effect of psychiatric disorders and smoking on white matter integrity. Zhang et al. (2010a), assessing patients with schizophrenia, demonstrated reductions in FA of the left anterior thalamic radiation/anterior limb of the internal capsule that were both independent and additive in smokers and patients with schizophrenia, such that smokers with schizophrenia had the largest reductions in FA. No studies to our knowledge have yet been conducted in patients with anxiety disorders.

In summary, gross and microstructural changes to key brain regions and white matter tracts are present in cigarette smokers and patients with anxiety disorders. Changes to white matter microstructure in certain regions connecting fear response areas have been associated with trait anxiety states, and it is possible that cigarette smoke could negatively affect these pathways. Future research into these areas may provide important insights into anxiety pathogenesis.

### Neurotransmitter systems

The importance of specific neurotransmitter systems has been extensively demonstrated in anxiety disorders, with current first-line pharmacological therapies interacting predominantly with the serotonergic, noradrenergic, cannabinoid, cholinergic, and dopaminergic systems. In addition, some of these agents are also effective in enhancing smoking cessation (Jorenby et al. 1999), suggesting a plausible biological interaction between these systems and nicotine dependence. Many studies have demonstrated that nicotine and cigarette smoke affect diverse neurotransmitter systems. However, how these may predispose to increased anxiety is very complex, involving interaction between systems and differing effects of cigarette components.

Much scientific work has explored the influence of nicotinic acetylcholine receptors (nAChRs) on brain function. The nAChRs are widely distributed throughout the central nervous system (CNS), located within synapses (pre and post synaptic), on cell bodies, dendrites, and axons (Bertrand 2010). Cholinergic innervation is widespread throughout the brain innervating nearly every neural zone. Many cholinergic projections do not terminate at synapses, but rather nonsynaptically where they contribute to diffuse volume transmission (Dani and Bertrand 2007). This could be the case for most hippocampal and cortical projections (Descarries et al. 1997), and would be similar to the action of other neurotransmitters including serotonin, dopamine, and noradrenaline (Vizi et al. 2010). What differentiates cholinergic transmission from these other neurotransmitters is that movement of acetylcholine (ACh) is via diffusion that is limited by acetylcholinesterase hydrolysis and not a reuptake pump (Dani and Bertrand 2007). Differently located nAChRs appear to exert different effects. Unlike in the periphery, where nAChR activation underpins fast neurotransmission at neuromuscular junctions, the role of fast transmission appears limited centrally although recent results suggest a possible role in hippocampal pyramidal neurons (Grybko et al. 2011). Many studies have identified a role for nAChRs in modulating neurotransmitter concentrations, with activation of presynaptic nAChRs known to enhance release of neurotransmitters acetylcholine, dopamine, noradrenaline, serotonin, glutamate, and gamma-aminobutyric acid (GABA) (Dani and Bertrand 2007). This appears to be consequence of facilitating increasing concentration of intracellular Ca^2+^ through augmenting calcium influx and altering activity of voltage-gated Ca^2+^ channels within the terminal. Alterations in multiple receptor regulated intracellular Ca^2+^ pathways are linked to mood and anxiety disorders (Plein and Berk 1999, 2001; Berk et al. 2001).

Numerous investigations have explored how activation of nAChRs by nicotine can exert effects on mood and anxiety symptoms. Nicotine can lead to both anxiogenic and anxiolytic effects that appear to depend upon the animal strain, dosing regimen, and experimental paradigm utilized. Exposure to nicotine in rat models leads to upregulation of nAChRs (Slotkin 2004) that is shortly followed by desensitization as exposure is continued. Activation of nAChRs, particularly the α4β2 and α7 subtypes, appears to enhance release of serotonin in several brain regions, including the dorsal raphe nucleus (Reuben and Clarke 2000; Ma et al. 2005). Interestingly, α4β2 receptor knockout mice demonstrate an increase in basal anxiety (Ross et al. 2000), suggesting a role for these receptors in anxiety regulation. These receptors also upregulate dopamine and noradrenergic neurons (Lichtensteiger et al. 1988), with these effects likely important in mediating the anxiolytic effects of nicotine (McGranahan et al. 2011). Data from a human study demonstrated that cigarette smoking upregulated dopamine release from the limbic system, a finding that correlated with improvement in mood and anxiety symptoms, although the improvement in anxiety symptoms didn't appear dependent upon nicotine (Brody et al. 2009). Acute activation of nAChRs by nicotine appears to produce anxiolytic effects in mouse models that can be blocked by nAChR antagonist mecamylamine. In addition, nicotine appeared to attenuate expression of c-Fos in numerous brain areas normally upregulated during stress, including the paraventricular hypothalamic nucleus, lateral hypothalamus, central amygdaloid nucleus, medial amygdaloid nucleus and cingulate and retrosplenial cortices (Hsu et al. 2007). In one controlled study conducted in humans, administration of nicotine also improved mood in nonsmokers with major depression (McClernon et al. 2006). In contrast to these findings, acute administration of nicotine into the lateral septum of rats precipitated an anxiogenic effect that was at least partially mediated by serotonin 1A receptors (Cheeta et al. 2000). Enhanced anxiety is a known initial side effect to the early administration of selective serotonin reuptake inhibitors (SSRIs) (Spigset 1999), a time of significantly increased serotonergic transmission. It is possible that enhanced release of serotonin via nAChR activation may partially explain nicotine's anxiogenic effects in some circumstances. It should be noted, however, that acute effects of nicotine generally appear to differ from chronic effects, with homeostatic adaptations potentially underpinning longer term effects.

In this context, the above results suggesting an acute anxiolytic effect of nicotine in animal models contrasts sharply with knowledge that most available antidepressants are antagonists of nAChRs (Shytle et al. 2002) and physostigmine, a potent acetylcholinesterase inhibitor, produces increased depressive and anxiety symptoms when administered (Janowsky et al. 1974). A further observation that may help clarify these seemingly conflicting effects is that of nicotine-induced nAChR desensitization. Desensitization of nAChRs is a complex process that occurs with normal cholinergic transmission and varies with degree of transmission and receptor subtype (Dani and Bertrand 2007). As nicotine enters the brain more gradually and is cleared more slowly than endogenous ACh, nicotine has the ability to induce more sustained desensitization of nAChRs (DeBry and Tiffany 2008). In this regard, exogenous nicotine can potentially exert a more profound inhibition of nAChRs than endogenous acetylcholine, leading to a potential decrease in release of various neurotransmitters. To support this, desensitization of nAChRs by low concentrations of nicotine lead to reduced release of GABA and dopamine in mice brains (Grady et al. 2012). These effects may underpin observations in human studies of depression, where nicotine and other cigarette components altering neurotransmitter system may partially explain development of depressed states (Dome et al. 2010). For example, smoking has been associated with impaired serotonin function as measured by fenfluramine challenge and measurement of colony-stimulating factor (CSF) levels of 5-hydroxyindoleacetic acid (Malone et al. 2003). However, this mechanism is likely more complex than a simple up- or downregulation of neurotransmitter release and responses vary with different nAChR subtypes. For example, long-term potentiation responses in the hippocampal CA1 region appear differentially affected by α7- and β2-containing nAChRs (Nakauchi and Sumikawa 2012). One factor that further complicates interpretation of this research relates to nicotine withdrawal, which is anxiogenic in animal and human studies (Picciotto et al. 2002). In this regard, anxiolytic effects of nicotine exposure may be secondary to relief of withdrawal (Mueller et al. 1998).

As it currently stands, the best explanation for how both agonism and antagonism of nAChRs may exert antidepressant and anxiolytic effects relates to desensitization. Direct exposure to nicotine can facilitate rapid desensitization of nAChRs, such that an indirect antagonist effect is rendered. This “functional antagonism” (Gentry and Lukas 2002) may underpin the antidepressant and anxiolytic effects of nicotine (Picciotto et al. 2008), although further research into the various effects of different nAChR subtypes and their relative activation/desensitization balance is required.

It is also important to consider how other components of cigarette smoke influence neurotransmitter function. Smoking exerts effects on monoamine oxidase (MAO) expression, including downregulation of MAO-A and MAO-B in the brain (Fowler et al. a,b) as well as influencing methylation of MAO promoter genes (Rendu et al. 2011). Free radicals, another highly concentrated component of cigarette smoke, can stimulate production of cell-mediated immune cytokines such as interferon-gamma (IFN-γ) (Nunes et al. 2012). These proinflammatory cytokines can influence serotonin metabolism, by activating indoleamine 2,3-dioxygenase to preferentially convert tryptophan into tryptophan catabolites, including kynurenine and quinolinic acid, in lieu of serotonin. This can precipitate a relative deficit in both tryptophan and serotonin, which has been, although not exclusively, associated with increased depressive and anxiety symptoms (Argyropoulos et al. 2004; Bell et al. 2005; Kulz et al. 2007).

### Inflammation and cell-mediated immune activation

Inflammation and activation of cell-mediated immune functions appears to be associated with psychiatric disorders (Dantzer et al. 2008; Miller et al. 2009; Wager-Smith and Markou 2011; Moylan et al. 2012b). Stress-induced inflammatory mediators may impair key brain processes in the hippocampus and PFC, including neuronal and synaptic plasticity, neurogenesis, long-term potentiation, and regulation of NTs. These actions may form part of anxiety disorder pathogenesis (for review see Hovatta et al. 2010) similar to their role in major depressive disorder (for reviews see Maes et al. 2011a; Moylan et al. 2012b). Cigarette smoking promotes increased systemic inflammation and cell-mediated immune reactivity, processes thought key to the pathogenesis of chronic physical disorders such as chronic obstructive pulmonary disease (Bhalla et al. 2009; Nikota and Stampfli 2012) and atherosclerosis (Ambrose and Barua 2004; Armani et al. 2009). These actions may also contribute to pathogenesis of anxiety.

Numerous studies have investigated levels of inflammatory mediators in anxiety disorders and increased anxiety states (Wadee et al. 2001). The results are heterogeneous, endorsing both increases and decreases in mediators. For example, psychological stress has been associated with increased production of proinflammatory cytokines including tumor necrosis factor-alpha (TNF-α), interleukin-6 (IL-6), interleukin-1 receptor antagonist (IL-1Ra), and IFN-γ, coupled with decreased production of anti-inflammatory cytokines including interleukin-10 (IL-10) and interleukin-4 (IL-4), with higher anxiety responses associated with significantly greater IFN-γ (Maes et al. 1998). In another study, clinically anxious individuals with a Hospital Anxiety and Depression Scale (HADS) score ≥8 demonstrated significantly higher levels of IL-6 and lower levels of serum cortisol, but no difference in C-reactive protein (CRP), compared with nonanxious individuals after controlling for depression and neuroticism (O'Donovan et al. 2010).

Others studies, however, have demonstrated an inverse relationship between psychological stress and levels of TNF-α (Chandrashekara et al. 2007). Studies in patients with OCD also demonstrate varying (Brambilla et al. 1997; Monteleone et al. 1998; Denys et al. 2004; Konuk et al. 2007) expression of plasma TNF-α, interleukin-1-beta (IL-1β), and IL-6. The first cytokine study performed in OCD found no increase in levels of interleukin-1 (IL-1), IL-6, or soluble interleukin-2 receptor (sIL-2R), although severity of compulsive symptoms was positively correlated with concentrations of plasma IL-6 and interleukin-6 receptor (IL-6R) (Maes et al. 1994), suggesting that IL-6 signaling may be associated with compulsive behavior. In another study comparing OCD and generalized social anxiety disorder (GSAD), lipopolysaccharide-induced production of IL-6 was decreased in OCD but maintained in GSAD (Fluitman et al. 2010). Interestingly, patients with OCD generally demonstrate lower rates of smoking than in other anxiety disorders (Bejerot and Humble 1999), with results also suggesting possible cholinergic supersensitivity in these disorders (Lucey et al. 1993).

Few studies have investigated inflammatory cytokines levels (Marazziti et al. 1992; Brambilla et al. 1999) and alterations of other immune cell markers (Rapaport 1998; Park et al. 2005) in PD, with data showing heterogeneous results. No significant changes in any of these variables could be found during CO_2_ inhalation-induced panic (van Duinen et al. 2008). Numerous investigations support upregulated inflammatory activity in PTSD (for review see Gill et al. 2009), including increased production of IL-6 (Maes et al. 1999; Bob et al. 2010), increased TNF-α, IFN-γ, IL-1β, and decreased production of interleukin-8 (IL-8) and interleukin-2 (IL-2). More recently, increases in a number of proinflammatory cytokines and chemokines (IL-6, IL-1α, IL-1β, IL-8, monocyte chemotactic protein-1 [MCP-1], macrophage inflammatory protein-1 α [MIP-1α], eotaxin, granulocyte macrophage CSF [GM-CSF], interferon-alpha [IFN-α]) were observed in patients with PD and PTSD (Hoge et al. 2009).

A number of factors may help explain the above heterogeneous results, including differences between anxiety disorder subtypes, study design, and confounding factors. Despite these heterogeneous results, other insights suggest increased inflammation contributes to anxiety pathogenesis. For instance, cytokine-based immunotherapy can lead to increased anxiety symptoms (Maes et al. 2001). Furthermore, depressive and anxiety symptoms induced by administration of cytokines are responsive to selective SSRIs (Gupta et al. 2006; de Knegt et al. 2011).

We are not aware of any studies that have assessed the impact of cigarette smoking on inflammatory mediator expression in anxiety disorders. However, there is evidence that smoking and depression act synergistically to increase inflammation (Nunes et al. 2012). Further, in a study assessing cytokine levels in the gingival crevicular fluid in patients with periodontal disease, levels of inflammatory cytokines IL-6 and IL-8 were both positively correlated with increasing psychological stress, as measured by the Modified and Perceived Stress Scale (Linn 1986), and cigarette smoking (Giannopoulou et al. 2003).

### Oxidative and nitrosative stress

Free radicals are by-products of oxidative phosphorylation that, at low or moderate concentrations, participate in normal cellular processes such as signaling pathways, mitosis, apoptosis, and responses to injury or infection (Valko et al. 2007). However, damage can occur to cellular components, including proteins, nucleic acid, carbohydrates, and lipids when levels of oxidative free radicals increase beyond the antioxidant capacity of cells. Increases in free radical concentrations can occur through both increased production of reactive oxygen species (ROS) and reactive nitrogen species (RNS) and/or decreased expression of antioxidants (Hovatta et al. 2010). Damage to these cellular components can alter the structure and function of membrane fatty acids and proteins, and alter or damage DNA and mitochondrial function leading to cell death (Maes et al. 2011b).

Increased plasma markers of O&NS have been repeatedly demonstrated in anxiety-disordered populations and animal models of anxiety (for review see Hovatta et al. 2010). In addition, increased hippocampal oxidative stress is anxiogenic (de Oliveira et al. 2007). For example, plasma markers of increased lipid peroxidation (malondialdehyde [MDA], thiobarbituric acid reactive substances [TBARS]) have been demonstrated in OCD (Ersan et al. 2006; Chakraborty et al. 2009; Ozdemir et al. 2009), SP (Atmaca et al. 2008), PD (Kuloglu et al. 2002), and PTSD (Tezcan et al. 2003). Further, studies in populations with anxiety disorders have demonstrated increased activity of antioxidant enzymes superoxide dismutase (SOD), catalase (CAT), xanthine oxidase, glutathione reductase (GSR), and glutathione peroxidase (Kuloglu et al. 2002; Tezcan et al. 2003; Herken et al. 2006; Atmaca et al. 2008; Ozdemir et al. 2009). Although these effects are not consistent across all studies (Ozdemir et al. 2009; Hovatta et al. 2010), it suggests that increased levels in oxidative stress do appear in anxiety-disordered populations. Psychological stress appears to be associated with increased O&NS and brain region specific O&NS induced cellular damage (Hovatta et al. 2010), as demonstrated by increased superoxide production in the mitochondria of rat hippocampus and PFC under chronic mild stress (Lucca et al. 2009), and increased NO production in rat hippocampus (Harvey et al. 2004) and in rat cortex (Olivenza et al. 2000) in a stress–restress animal model. Psychological stress (e.g., examination stress) is accompanied by an increase in inflammatory markers, lipid peroxidation, oxidative damage to DNA, and reduced antioxidant activity in the plasma (Wadee et al. 2001; Sivonova et al. 2004). Increased stress levels (e.g., increased perceived workload) and the impossibility to cope with stress have been associated with elevated 8-hydroxydeoxyguanosine (8-OHdG) levels (Irie et al. 2005).

ROS and RNS interact in a bidirectional fashion with proinflammatory cytokine signaling pathways (Hovatta et al. 2010) leading to enhanced O&NS. One example is of neopterin, which is synthesized from macrophages after stimulation by proinflammatory cytokines (IFN-γ). Production of neopterin increases production of NO through upregulating inducible nitric oxide synthase (iNOS) gene expression (Maes et al. 2012). Further, the proinflammatory cytokines IL-1β and TNF-α increase superoxide production by stimulating arachidonic acid release, leading to nicotinamide adenine dinucleotide phosphate (NADPH) oxidase activation (Chenevier-Gobeaux et al. 2007), and activation of proinflammatory transcription factors, including nuclear factor k β (NFkβ) and the cyclic adenosine monophosphate (cAMP) response element binding (CREB) family, appear to regulate the production of O&NS by modulating the activity of NOS, cyclooxygenase 2 (COX2), and NADPH oxidase (Hovatta et al. 2010). Alterations in activity of these particular enzymes have also been linked to anxiety behaviors. For example, enhanced anxiety resulted from the downregulation of NOS through administration of a NOS inhibitor in one study (Masood et al. 2009). However, these results were not replicated in another study, which demonstrated that downregulation of NOS by stimulation of serotonin 1A receptors (5-HT1aR) lead to an anxiolytic effect, mediated by an increase in CREB phosphorylation resulting from decreased NO production (Zhang et al. 2010b). Increased activation of CREB in the nucleus accumbens is associated with increased neuronal survival (Mantamadiotis et al. 2002) and has also been associated with reduced anxiety (Barrot et al. 2005). Inhibition of phosphodiesterase E2 (PDE2), which in turn inhibits activity of NADPH oxidase, reduces anxiety behavior associated with induced oxidative stress (Masood et al. 2009). Increased hippocampal NADPH oxidase 1 activity appeared to increase anxiety behavior in rats with adjuvant arthritis (Skurlova et al. 2011).

Subchronic oxidative stress may mediate anxiety responses through effects on NTs and enzymatic activity. Subchronic oxidative stress appears to induce downregulation of brain-derived neurotrophic factor (BDNF), glyoxalase 1 (GLO1), and GSR1 (Salim et al. 2011). BDNF is a critical brain NT and also acts as a potential antioxidant mediator (Lee and Son 2009; Chan et al. 2010). Local increases in GLO1 and GSR1 enzyme expression, whose functions include protection against dicarbonylglycation and production of glycation end products (Hambsch 2011), have previously been associated with increased anxiety-like behaviors (Hovatta et al. 2005). However, Salim et al. (2011) demonstrated that subchronic oxidative stress downregulates GLO1 and GSR1 via induction of calpain expression in the hippocampus, predisposing to increased protein glycation and subsequent further oxidative stress. This increased oxidative stress, in concert with calpain activation (Shumway et al. 1999), is proposed to induce NFĸB transcription, leading to enhanced production of proinflammatory cytokines (IL-1, CRP, TNF-α) and inflammatory-mediated cellular damage (Salim et al. 2011). The induction of calpain mediated decreased expression of BDNF (see section Neurotrophins below) (Salim et al. 2011).

Cigarette smoke, a significant source of exogenous free radicals (Stedman 1968), contains thousands of chemicals that increase O&NS, and smokers or those exposed to passive smoke appear to have significantly reduced circulating antioxidants (Sobczak et al. 2004; Swan and Lessov-Schlaggar 2007). Many studies have demonstrated changes consistent with increased O&NS in the brains of animals exposed to cigarette smoke. Such changes include increased levels of ROS (Luchese et al. 2009) and RNS including superoxide, TBARS, carbonylated proteins (Tuon et al. 2010), measures of lipid peroxidation (Anbarasi et al. 2005a; Stangherlin et al. 2009; Thome et al. 2011), and reduction of antioxidant enzymes (Stangherlin et al. 2009) including SOD (Luchese et al. 2009), catalase (Luchese et al. 2009), glutathione peroxidase, GSR, glutathione, and vitamins (A, C, E) (Anbarasi et al. 2006a). It should be noted that there are some exceptions to this trend (Delibas et al. 2003; Fuller et al. 2010). Although some studies report an increase in these antioxidant enzymes after acute exposure to cigarette smoke (Baskaran et al. 1999), this is likely an adaptive response designed to protect against oxidative damage (Hilbert and Mohsenin 1996). Over the longer term, chronic cigarette exposure appears to overwhelm these adaptive host antioxidant responses (Hulea et al. 1995; Anbarasi et al. 2006a) leaving the system vulnerable to cellular damage. The importance of deterioration in antioxidant levels is underlined by the fact that cigarette smoke-induced increases in markers of lipid peroxidation are prevented by vitamin E (Thome et al. 2011). Furthermore, another study demonstrated that active exercise reduced expression of oxidative stress produced secondary to cigarette smoke exposure in rats (Tuon et al. 2010). The ability of exercise to modulate oxidative stress may also partially underpin its therapeutic effect on anxiety disorders (Moylan et al. 2013).

Exogenous nicotine administration to isolated cell lines in vitro reduces antioxidant constituents (e.g., glutathione) and increases markers of lipid peroxidation (MDA) and lactate dehydrogenase activity (Yildiz et al. 1998, 1999), effects blocked by addition of detoxifying enzymes SOD and CAT (Yildiz et al. 1998, 1999). Investigations into the effects of nicotine on oxidative stress in CNS cells have been more limited. In a study that utilized chronic nicotine exposure administered for 10 days, results demonstrated increased levels of TBARS and HNE (4-hydroxynonenal) in the brain (Bhagwat et al. 1998). Cigarette smoke can also increase levels of brain heat shock protein 70 kDa (Anbarasi et al. 2006b).

Only one study to our knowledge has simultaneously assessed the association between cigarette smoke exposure, anxiety symptoms, and brain oxidative stress markers. In this study, rats exposed to cigarette smoke showed increased markers of brain lipid peroxidation and decreased plasma ascorbic acid. When rats were additionally treated with pecan nut shell extract, a substance with antioxidant properties, improvements were demonstrated in anxiety symptoms (interpreted as withdrawal symptoms) and markers of lipid peroxidation (Reckziegel et al. 2011).

### Mitochondrial function

Mitochondria are important sources of oxidative stress and many abnormalities in mitochondrial function have been found in psychiatric disorders (for review see Manji et al. 2012). Although still requiring much investigation, multiple factors support a role for mitochondrial dysfunction in increasing anxiety. First, patients exhibiting mitochondrial disorders commonly demonstrate psychiatric symptoms including increased anxiety (Miyaoka et al. 1997; Anglin et al. 2012). Second, recent investigations have discovered decreased levels of glycolysis enzymes coupled with increased expression of components associated with the electron transport chain in high-anxiety trait animal models, potentially increasing vulnerability to production of ROS and subsequent cellular damage (Filiou et al. 2011). These results were coupled with observations of altered levels of proteins associated with neurotransmission in high-anxiety mice thought to be consequent to mitochondrial protein alteration (Filiou et al. 2011). The authors hypothesize that mitochondria may underpin a “unifying link between energy metabolism, oxidative stress, and neurotransmission alterations” that were observed between high- and low-anxiety trait mice. Third, mitochondria-targeted antioxidant SkQ1 has been associated with decreased expression of anxiety behaviors in rats (Stefanova et al. 2010). Finally, mutant mice with reduced function of Bcl-2, a key modulator of mitochondrial function, demonstrate increased anxiety behavior (Einat et al. 2005).

Exposure to cigarettes can lead to mitochondrial dysfunction (Miro et al. 1999; Anbarasi et al. 2005b), as demonstrated by increased levels of cholesterol, lipid peroxides and increased cholesterol/phospholipid ratio, in conjunction with decreased mitochondrial enzymes in those exposed to cigarette smoke. However, chronic cigarette smoking was not associated with derangement of mitochondrial function in a separate study, but did prevent exercise-induced improvement in mitochondrial function (Speck et al. 2011). A potential explanation for absence of demonstrable mitochondrial dysfunction in this study may relate to the use of SWISS mice in the experimental design that were demonstrated to be highly resistant to cigarette smoke-induced oxidative stress (Rueff-Barroso et al. 2010). Recent evidence suggests that nicotine exposure may worsen mitochondrial function through direct effects on membrane potential and granularity of desensitizing α7 nAChRs (Gergalova and Skok 2011). Given these preliminary results, investigation of therapies that promote mitochondrial function in patients with anxiety disorders would be fruitful. These studies should take in account smoking status.

### Neurotrophins and neurogenesis

Increasing evidence supports a role for NTs and neurogenesis in development of anxiety disorders and anxiety symptoms, although certain mediators may exert varying effects on different anxiety symptoms. Animal models have demonstrated stress-related changes to neurogenesis in areas associated with mood and anxiety disorders including the hippocampus (Cirulli et al. 2010). Exposure to neonatal stress can reduce expression of hippocampal BDNF via altering gene expression (Roth et al. 2009; Roth and Sweatt 2011), which may facilitate vulnerability to mood and anxiety as consequence of decreased neuronal survival (Gomez-Pinilla and Vaynman 2005). In addition, altered levels of BDNF and their Trk B receptors may occur in dopaminergic pathways projecting from the ventral tegmental area in the midbrain to the nucleus accumbens (Yu and Chen 2011). Changes in BDNF appear associated with increased anxiety behaviors. Intrahippocampal injections of BDNF in rats lead to an increase in anxiety assessed by facilitatory avoidance and the light–dark test. This was blocked by a 5HT1a antagonist suggesting a modulatory role of serotonin (Casarotto et al. 2012). Social deprivation stress leads to the development of anxiety in mice, and this appears to be modulated by reductions in BDNF (Berry et al. 2012). In a cross-sectional study of a healthy population, plasma BDNF levels were negatively associated with somatization, obsessive–compulsiveness, interpersonal sensitivity, and anxiety (Bhang et al. 2012). BDNF may also be a modulatory factor in the development of PTSD (Rakofsky et al. 2012).

Another NT that appears important in anxiety regulation is nerve growth factor (NGF). NGF is increased under conditions of stress in both animal models and humans (Aloe et al. 1986, 1994, 2002), and appears to be important in resilience to stress-related neuropsychiatric disorders (for review see Alleva and Francia 2009). Interestingly, animal models demonstrate that increases in release of NGF are most marked under conditions of stressful behavioral interactions between animals, with lesser increases seen under physical restraint stress (Aloe et al. 1986; Branchi et al. 2004; Alleva and Francia 2009). Further evidence suggests that levels of fibroblast growth factor 2 (FGF2) in the hippocampus are decreased in animals with higher anxiety and lower response to novelty (Perez et al. 2009) and that early life administration of FGF2 is able to prevent increased anxiety in later life (Turner et al. 2011).

Maternal exercise can lead to increased expression of NTs, including VEGF and BDNF, in the PFC of offspring that is associated with decreased anxiety (Aksu et al. 2012). Exercise also appears able to protect against the negative effect of maternal deprivation on expression of these NTs (Uysal et al. 2011).

Cigarette smoking and nicotine in particular appear to exert effects on expression of NTs, although the literature is sparse and heterogeneous. For example, cigarette smoking and repeated nicotine exposure has been associated with decreased expression of BDNF in animal models (Yeom et al. 2005; Tuon et al. 2010). In addition, plasma levels of BDNF are significantly lower in smokers than nonsmokers in human studies, with levels increasing with greater duration of smoking abstinence (Kim et al. 2007; Bhang et al. 2010). However, other results have suggested that nicotine exerts a positive effect on BDNF levels. For example, nicotine administration has been associated with increased levels of BDNF and FGF-2 in animal striatum (Maggio et al. 1997). The neurotrophic augmenting effects of nicotine in this situation is hypothesized to underpin a therapeutic benefit of cholinergic stimulation on Parkinson's disease by protecting dopaminergic neurons from damage. In a further study, traumatic brain injury revealed a positive effect of chronic cigarette smoking on BDNF expression (Lee et al. 2012). Nicotine exposure has also been associated with significant increases in NGF (French et al. 1999; Hernandez and Terry 2005) in the hippocampus and with transient decreases in NT-3 (French et al. 1999), although once again results are not consistent which may relate to differences in nicotine administration (Hernandez and Terry 2005).

Differences in NT expression in response to cigarette smoking are likely dependent upon numerous factors, including the relative roles of nicotine and other components of cigarette smoke (e.g., free radicals) and the developmental stage at which exposure occurs. Given the key role of NTs in brain neurodevelopment, distortion to different NTs in early development may facilitate disordered growth in brain architecture (Abreu-Villaca et al. 2003a; DeBry and Tiffany 2008). Such effects may leave the overall system more vulnerable to disorders such as increased anxiety. If exposure occurs later, alterations to NTs may undermine normal compensatory and protective mechanisms available to neuronal cells, leaving cells at greater risk of damage or induced apoptosis. Future studies should evaluate the roles of nicotine and other constituents of cigarette smoke on the levels of NTs correlated with anxiety and depressive behaviors in animal models, taking into account the different stages of development at which exposure can occur.

### Epigenetic effects

The study of epigenetic changes in anxiety disorders is a relatively new field, although some preliminary evidence suggests that cigarette smoke may lead to changes in gene expression predisposing to increased anxiety. For example, smoking has been associated with epigenetic regulation of MAO-B via a reduction in methylation of its gene promoter. This change leads to increased production of MAO-B persisting long after smoking is ceased (Launay et al. 2009) that can alter neurotransmitter concentrations. In addition, prenatal exposure to environmental tobacco smoke has been demonstrated to modify expression of genes controlling key functions such as synaptic function, neurogenesis, axonal growth, and cellular survival in the developing hippocampus (Mukhopadhyay et al. 2010). Data from cardiovascular research have also demonstrated the potential of gestational cigarette smoke exposure to upregulate expression of genes associated with production of proinflammatory substances in developing primates, which may increase vulnerability to vascular disease in later life (Villablanca et al. 2010). In depression, preliminary research has identified interrelationships between levels of gene methylation and inflammatory mediators that may contribute to pathogenesis via alteration of tryptophan metabolism (Uddin et al. 2011). Investigation of epigenetic changes may provide insights into how cigarette smoking can impact gene expression in potentially contributing to pathogenesis of anxiety disorders, although empirical data are currently very limited. One potential genetic influence that could be explored is the role of prototoxin gene LYNX2. LYNX2 encodes for proteins that modulate activity of neuronal nAChRs, the neural target of smoking-ingested nicotine. LYNX2-encoded proteins modify nAChR receptor control of glutamate release from the medial PFC. Loss of LYNX2 was associated with increased glutamatergic activity and increased anxiety behaviors in one study, suggesting a possible role in controlling anxiety responses (Tekinay et al. 2009). Further studies are required to assess whether LYNX2 functioning may affect the alterations to nAChRs provoked by prolonged nicotine exposure in smokers.

**Figure 1:**
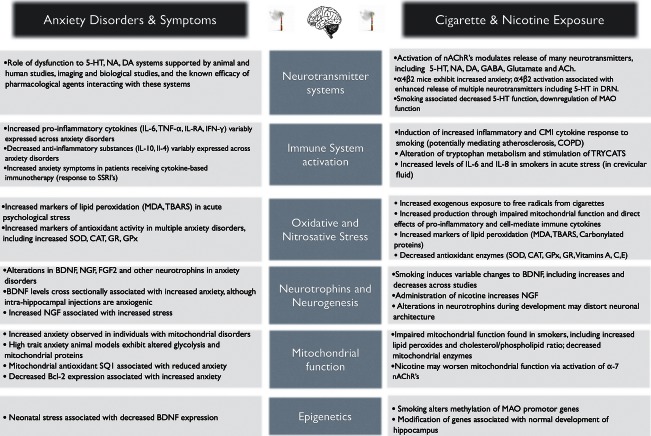
Multiple pathways that are associated with development of anxiety disorders are affected by cigarette smoke and nicotine, including diverse neurotransmitter systems, inflammation and the immune system, oxidative and nitrosative stress, neurotrophins and neurogenesis, mitochondrial function, and epigenetic influences. It is possible these pathways may underpin how exposure to cigarette smoke could increase anxiety symptoms and expression of anxiety disorders. 5-HT, 5-hydroxytryptophan; BDNF, brain-derived neurotrophic factor; CAT, catalase; CMI, cell-mediated immune; DA, dopaminergic; DRN, dorsal raphe nucleus; FGF2, fibroblast growth factor 2; GPX, glutathione peroxidase; GSR, glutathione reductase; IFN-γ, interferon-gamma; IL-1, interleukin 1; IL-1RA, interleukin 1 receptor antagonist; IL-10, interleukin-10; IL-12, interleukin-12; IL-6, interleukin 6; MAO, monoamine oxidase; MDA, malondialdehyde; NA, noradrenergic; NGF, nerve growth factor; O&NS, oxidative and nitrosative stress; PICs, proinflammatory cytokines; SOD, superoxide dismutase; SSRI, selective serotonin reuptake inhibitor; TBARS, thiobarbituric acid reactive substances; TNF-α, tumor necrosis factor-alpha; TRYCATs, tryptophan catabolit.

The above findings (summarized in Fig. 1) suggest a potential role for inflammation, O&NS, mitochondria, NTs, and epigenetic alterations in the pathogenesis of anxiety disorders, although further investigation is required to delineate these relationships. Cigarette smoking can modulate all of these pathways, potentially distorting cellular functioning and neuronal architecture predisposing to higher vulnerability to developing anxiety disorders.

## Cigarette Smoke Exposure, Nicotine, and Altered Neurodevelopment: Increasing the Risk of Anxiety Disorders?

Cigarette smoke is known to be deleterious to neurodevelopment (Picciotto et al. 2002; Slotkin 2004; Slikker et al. 2005; DeBry and Tiffany 2008), and exposure to cigarette smoke in early neurodevelopment appears to increase the risk of developing anxiety in later life (Bandiera et al. 2011; Jamal et al. 2011). During early neurodevelopment, cigarette exposure can be direct (e.g., early adolescent smoking, in utero exposure to maternal smoking), or second hand as environmental smoke exposure (Bandiera et al. 2011). Given the diversity of active compounds in cigarette smoke, we focus here primarily on the specific influence of nicotine (Newman et al. 2002a) on neurodevelopment. However, as cigarette smoke contains many substances that either directly (e.g., free radicals) or indirectly (e.g., metals) exert effects on O&NS stress pathways, immune and mitochondrial functions, it is possible these effects also influence neurodevelopment and potentially subsequent anxiety risk.

Nicotine readily crosses the placenta and enters the fetal blood stream in utero (Lambers and Clark 1996). Exposure in utero has also been associated with later behavioral and social problems (Nicoll-Griffith et al. 2001; Piquero et al. 2002; Brion et al. 2010), suggesting the potential to alter neurodevelopmental trajectories. Nicotine's action as a specific agonist of nAChRs is facilitated by the very early expression (prior to neurulation) of these receptors in the developing CNS (Atluri et al. 2001; Schneider et al. 2002). Cholinergic inputs are critical during brain development, underwriting many key processes including axonal and synaptic growth, promoting neurogenesis, facilitating planned apoptosis, and initiating the switch between neuronal replication and differentiation (Slotkin 2004). Increasing evidence suggests nicotine agonism of nAChRs may exert deleterious neurodevelopmental effects. Given the rapid rate of neurodevelopment in utero, deleterious effects will likely be most significant during this time, but might continue to occur at any time when there is significant development (e.g., during adolescence) (DeBry and Tiffany 2008).

Agonism of nAChRs by nicotine is more prolonged than that exerted by acetylcholine in normal cholinergic transmission due to differences in concentration and clearance. Nicotine presented in utero is usually present in higher concentrations, and is more slowly cleared, than endogenous acetylcholine (DeBry and Tiffany 2008). As consequence, nicotine can induce enhanced nAChR activation, facilitating adaptive effects such as receptor desensitization, and if excessive, direct toxicity (DeBry and Tiffany 2008). These “neuroteratogenic” effects occur at doses well below that required for growth impairment. Animal models have demonstrated that nicotine exposure leads to quite profound distortion of early neural development characterized by increased apoptosis and enlargement of intercellular spaces (Roy et al. 1998). Even though substantial recovery appears to occur such that brains with grossly distorted architecture in utero do not appear grossly abnormal in adolescence or adulthood, there remain long-lasting effects of nicotine exposure to neuronal architecture, cellular functioning and survival, and DNA expression and regulation. For example, prenatal nicotine exposure has been associated with persisting alterations in cellular architecture in the hippocampus (Roy et al. 2002; Abdel-Rahman et al. 2005; Oliveira-da-Silva et al. 2009) and somatosensory cortex (Roy and Sabherwal 1994), decreased DNA expression in brainstem, forebrain, and cerebellum (McFarland et al. 1991), persistently enhanced markers of cellular damage and apoptosis (e.g., c*-fos* and ornithine decarboxylase; Slotkin et al. 1991; Trauth et al. 1999). Many effects have demonstrated persistence into adolescence. For example, decreased synaptic activity in noradrenergic and dopaminergic neurons evident in the early postnatal period of rats exposed to nicotine prenatally has been demonstrated to reemerge with pubertal onset (Navarro et al. 1988), either as reduced activity or as reduced responsiveness to normal cholinergic stimulation (Seidler et al. 1992). Prenatal exposure to nicotine also causes ongoing alteration to nAChRs that extend in adolescence (Gold et al. 2009). In addition, genetic profiling studies have revealed that adolescent genes coding for the ventral tegmental area, some of which encode for neurite development, psychological disorders, development disorders, and nervous system development, appear more vulnerable to long-term effects of chronic nicotine exposure than adult genes (Doura et al. 2010).

Evidence supports that exposure to nicotine prenatally and during early postnatal life leads to increased anxiogenic behaviors in rats (Eppolito et al. 2010). Interestingly, measures of increased anxiety behavior in the *Elevated Plus Maze* in those rats exposed to prenatal nicotine were present in adulthood but not in adolescence, and although the result was more prominent in female rats, males also demonstrated the response (Eppolito et al. 2010). The exposure to nicotine before and shortly after birth was associated with impairment to fear extinction (Eppolito et al. 2010), which replicated results from chronic nicotine exposure in adolescence but not adulthood (Smith et al. 2006). This may suggest that exposure to nicotine in high-activity neurodevelopmental periods may exert more deleterious effects than in adulthood. It is possible that chronic administration of nicotine, via altered nAChR activity, may influence gene expression and plasticity in the medial PFC and amygdala (Brown and Kolb 2001; Li et al. 2004; Polesskaya et al. 2007). This interaction may underpin the lack of extinction learning displayed in rats that are exposed to chronic nicotine (Eppolito et al. 2010).

Nicotine induces production of oxidative stress markers and reduces antioxidant defenses, contributing a major proportion of the net oxidative stress from cigarette use (Bhagwat et al. 1998; Yildiz et al. 1998; Guan et al. 2003; Qiao et al. 2005; Das et al. 2009), although nicotine is known exhibit both pro- and antioxidant effects (Li et al. 2000; Tizabi et al. 2003). Nicotine increases lipid peroxidation markers that can be prevented by coadministration of free radical scavenger vitamin E (Qiao et al. 2005) and has demonstrated antimitotic properties (Qiao et al. 2003). Increased production of O&NS, and antimitotic properties, has been demonstrated during cell differentiation (in association with increased in nAChR density) (Qiao et al. 2003, 2005). It is possible that the balance between damaging and protective effects of nicotine may depend upon the degree of stimulated oxidative stress – a small amount of oxidative stress could have positive effects in stimulating normal cellular processes, but significantly increased oxidative stress could overwhelm protective mechanisms leading to direct cellular damage (Newman et al. 2002a).

Given the increased level of O&NS present in adolescence, it could be hypothesized that vulnerability to toxic effects of nicotine-induced oxidative stress would be heightened (Qiao et al. 2005). Nicotine has demonstrated adverse neurobiological effects during adolescence, with these effects seemingly dependent on only early small and infrequent exposure to nicotine (Abreu-Villaca et al. 2003b). In keeping with this hypothesis, administration of nicotine for 1 week to adolescent rats resulted in a significant increase in TBARS with effects that would have been observed at low levels of exposure (Qiao et al. 2005).

Other mechanisms of nicotine-induced damage may include upregulation of mRNA expression encoding proteins associated with cell death and cell differentiation. For example, increased levels of such proteins (p53) and reduction of DNA were found in the hippocampus, cortex, and midbrain in adolescent rats (Trauth et al. 2000). These effects were not of the same magnitude as those seen with nicotine exposure in utero (Levin and Slotkin 1998).

Nicotine also exerts effects on numerous trophic factors (DeBry and Tiffany 2008; Son and Winzer-Serhan 2009), including upregulation of FGF (Belluardo et al. 2004), PDGF (platelet-derived growth factor), BDNF (Kenny et al. 2000), Trk A (Formaggio et al. 2010), and NGF. It is possible that exacerbated expression of these growth-supporting factors via nicotine's agonism of nAChRs may interfere with normal neurodevelopmental processes. As nicotine's stimulation of nAChRs is potentially more prolonged than normal cholinergic transmission, expression of NTs may be higher than required for normal neurodevelopment, with this higher expression leading to disordered development of neuronal architecture (Abreu-Villaca et al. 2003c). Such effects may predispose an increased risk of developing anxiety and other psychiatric disorders in later life.

## Therapeutic Implications for Anxiety Disorders

A number of these insights may have treatment implications for anxiety-based disorders and symptoms. It is hypothesized that adaptation and desensitization of nAChRs may underpin the effect of cigarettes on anxiety and mood regulation (Mineur and Picciotto 2010), based on the association between higher smoking rates and mood dysregulation (e.g., depression) (Covey et al. 1998), animal models demonstrating antidepressant effects of acute nicotine on learned helplessness (Semba et al. 1998) and other depression behaviors (Djuric et al. 1999; Tizabi et al. 1999), the effect of antidepressants such as bupropion as smoking cessation aids (Hurt et al. 1997) and that some antidepressants also serve as noncompetitive inhibitors of nAChRs (Shytle et al. 2002). Many of these effects apply to increased anxiety, suggesting that certain central nAChRs may serve as a new potential treatment target.

Numerous studies have demonstrated potential for use of centrally acting nAChR antagonists in anxiety treatment. For example, the nAChR antagonist mecamylamine has produced anxiolytic improvement in multiple animal models (Newman et al. 2002b). Mecamylamine was demonstrated to be a useful augmentation agent to SSRI treatment of major depression (George et al. 2008), and administration of mecamylamine also appears capable of blocking dexamethasone-induced anxiety, which occurs concurrently with upregulation of BDNF levels (Park et al. 2011). The anxiolytic effects of nAChR antagonism have also been confirmed using an alternative agent, lobeline (Roni and Rahman 2011). Human data on the use of nAChR antagonist for anxiety are scarce. A possible insight comes from the use of bupropion, which blocks reuptake of noradrenaline and dopamine but in addition exerts a noncompetitive antagonist effect at several subtypes of nAChRs (Arias 2009). Bupropion has demonstrated significant anxiolytic effects equivalent to the action of SSRIs in treatment of patients with major depressive disorder, and this effect on nAChRs may underpin part of this effect, although other explanations are possible (e.g., effect on noradrenergic and dopaminergic systems) (Papakostas et al. 2008). Future randomized studies will provide insight into therapeutic possibilities exploiting modification of nAChRs in treating anxiety disorders.

Other potential agents worthy of consideration include agents that can guard against inflammatory- and O&NS-mediated effects. For example, the tetracyclic antibiotic minocycline, which exerts strong anti-inflammatory effects, has been shown to potentially modulate anxiety behaviors after cardiac arrest (Neigh et al. 2009). In addition, inhibition of COX2 has been shown in animal models to prevent anxiety development (Casolini et al. 2002), and celecoxib, a COX-2 inhibitor, has shown benefit as an augmentation agent to SSRIs in depression (Muller et al. 2006). Antioxidant treatments, such as *n*-acetylcysteine, have shown some promise in augmentation of treatment in mood disorders (Berk et al. 2012), and exploration of their utility in anxiety disorders would be of use.

Further research exploring the effects of agents that influence the identified pathways may provide important new avenues for therapy for pathological anxiety. In addition, such work will be important in further understanding the biological pathways facilitating development and reinforcement of cigarette smoking. These efforts may assist the development of more advanced treatments for smoking cessation, particularly in patients with anxiety disorders who exhibit poor rates of success to traditional cessation strategies (Piper et al. 2011).

## Limitations and Directions for Future Research

A number of interpretational caveats must be considered when considering the evidence presented. First, the literature discussed above is drawn from a variety of sources, utilizing differing measures of anxiety (e.g., different diagnostic measures for individual anxiety disorders, different symptoms scale measures of anxiety or psychological stress, different animal anxiety models) and different rates of smoking or nicotine exposure. Studies also employed various confounders and other study designs which make cross-comparisons difficult. Where possible, we have discussed individual anxiety disorder diagnoses noting the significant distinctions between different disorder groups. In particular, evidence suggests that different anxiety disorder subtypes display significantly different rates of smoking (Kalman et al. 2005). However, the scant literature available for some pathways prevented an analysis by disorder subtype, with most evidence on potential pathway effects drawn from cross-sectional investigations of animal models. The cross-sectional nature of this literature impeded conclusions regarding causation, and it is possible that observations highlighted (e.g., smoking with changes to brain volume) may be unidirectional, bidirectional, or mediated by other shared factors. In addition, there currently exists a paucity of research assessing a particular pathway in concert with smoking and anxiety. Few prospective data are available assessing the impact of changes to specific systems on anxiety symptoms in response to cigarette smoking. In addition, aside from the inherent difficulties in translating animal model data to humans, many of the above associations displayed variability in results depending upon study variables, including animal model used or experimental design. In addition, much of the literature has focused solely on the role of nicotine and not the other known toxic ingredients of cigarette smoke including free radicals and metals. There was also significant variability in expression and function of these systems between different groups (e.g., men vs. women) and individuals within these groups, and hence much further work is required to ascertain how these influences draw together. Understanding reasons underpinning differential expression between groups may help clarify further key elements to anxiety development. Women, for example, are known to exhibit higher rates of anxiety disorders, which likely relates to a combination of biological (e.g., different hormonal compositions) and psychological factors, and hence further analysis of these effects on the described pathways may prove enlightening. The acute and long-term effects of any agent that causes a robust homeostatic adaptation are often quite different; this needs to be taken into account in interpretation of acute data, and in extrapolating to management strategies.

Future research efforts in this area should attempt to address some of these challenges. First, it would be useful to ascertain the effects of nicotine versus other cigarette constituents to the above pathways in humans. The use of populations with high consumption of Snus, such as Norway, presents as opportunity for such analyses to be conducted prospectively, and combined with follow-up behavioral assessments, serum analysis of relevant markers (e.g., inflammatory or O&NS), assessment of genetic function, and functional and structural imaging. Such studies could be extended over time to investigate specific changes between different anxiety disorders (e.g., PD, GAD, PTSD), different subsets of the population (e.g., cultural or gender groups), and in individuals with other risk elements known to influence these similar pathways (e.g., history of childhood trauma, comorbid medical illness). It is likely that interindividual differences in genetics and epigenetic alterations will also complicate these effects, and as such further exploration of this evolving area will be of foremost importance. Given that effects on multiple pathways may exert incremental increases in risk for developing anxiety, triangulation of potential effects involving a combination of animal and human models will likely be required as power to detect small effects will only be found in very large studies.

## Conclusion

Many studies have suggested that cigarette smoking may increase the risk of developing increased anxiety, although confirmation of this causality is yet to be confirmed. Evidence into pathogenesis of anxiety disorders and increased anxiety symptoms potentially supports a role for diverse neurotransmitter systems, the immune system, O&NS, mitochondrial function, and epigenetic regulation, although the literature is heterogeneous and scant in certain areas. Ingredients that are present in cigarette smoke, including nicotine and other toxic chemicals, exert influences on all of these pathways. These effects may at least partially underpin the biological mechanisms through which smoking may contribute to increased anxiety, and potentially serve as a useful framework for further research efforts. Similar pathways are likely to be operative in other states characterized by fight, flight, freeze responses such as anger, mood disorders (e.g., depressive states), and psychotic disorders. The exposure to nicotine and other cigarette ingredients may also exert neurodevelopment influences capable of changing anxiety trajectories, underscoring the importance of reducing exposure to cigarette during gestation and throughout childhood. Centrally, nAChRs appear to be a crucial mediator of the anxiety-modifying effects of cigarette smoke and may represent a future therapeutic target for anxiety disorders. In addition, anti-inflammatory and antioxidant agents may assist in improving anxiety symptoms, as they may do in depression. Further studies addressing this area may elicit insights into new therapeutic opportunities.
